# The Current Status of the Liver Liquid Biopsy in MASH Related HCC: Overview and Future Directions

**DOI:** 10.3390/biom13091369

**Published:** 2023-09-09

**Authors:** Onyinye Ugonabo, Utibe-Abasi Sunday Udoh, Pradeep Kumar Rajan, Heather Reeves, Christina Arcand, Yuto Nakafuku, Tejas Joshi, Rob Finley, Sandrine V. Pierre, Juan Ramon Sanabria

**Affiliations:** 1 Department of Medicine, Marshall University School of Medicine, Marshall University, Huntington, WV 25701, USA; ugonabo@marshall.edu (O.U.); tjoshi@marshall.edu (T.J.); 2 Marshall Institute for Interdisciplinary Research, Marshall University School of Medicine, Huntington, WV 25703, USA; udohu@marshall.edu (U.-A.S.U.); rajan@marshall.edu (P.K.R.); nakafuku@marshall.edu (Y.N.); pierres@marshall.edu (S.V.P.); 3 Department of Surgery, Marshall University School of Medicine, Marshall University, Huntington, WV 25701, USA; reevesh@marshall.edu (H.R.); arcand@marshall.edu (C.A.); finleyr@marshall.edu (R.F.); 4 Department of Nutrition and Metabolomic Core Facility, Case Western Reserve University School of Medicine, Cleveland, OH 44106, USA

**Keywords:** biological markers, HCC, liver, MASH

## Abstract

Metabolic dysfunction-associated steatohepatitis (MASH) is one of the major risk factors for chronic liver disease and hepatocellular carcinoma (HCC). The incidence of MASH in Western countries continues to rise, driving HCC as the third cause of cancer-related death worldwide. HCC has become a major global health challenge, partly from the obesity epidemic promoting metabolic cellular disturbances but also from the paucity of biomarkers for its early detection. Over 50% of HCC cases are clinically present at a late stage, where curative measures are no longer beneficial. Currently, there is a paucity of both specific and sensitive biological markers for the early-stage detection of HCC. The search for biological markers in the diagnosis of early HCC in high-risk populations is intense. We described the potential role of surrogates for a liver biopsy in the screening and monitoring of patients at risk for nesting HCC.

## 1. Introduction

Primary liver cancer comprises two major histological types, hepatocellular (HCC) and intrahepatic cholangiocarcinoma (ICC). HCC accounts for 90% of primary liver cancers, with more than 840,000 cases globally [1,2]. Risk factors for the development of HCC include, among others-chronic hepatitis B virus (HBV), chronic hepatitis C virus (HCV), metabolic dysfunction-associated steatotic liver disease (MASLD), alcohol-related liver disease (caused by excessive alcohol consumption), cirrhosis, hemochromatosis, primary sclerosis cholangitis, primary biliary cirrhosis, autoimmune hepatitis, Wilson’s disease, Alpha-1 antitrypsin deficiency, dietary aflatoxins and algal hepatotoxins in water. HBV and HCV are being displaced as the leading cause by the inflammatory evolution of MASLD (metabolic dysfunction-associated steatohepatitis or MASH) [3]. HCC is known for its poor prognosis, probably because >50% of patients are diagnosed at a late stage. Early diagnosis is critical for having access to effective therapeutic options such as liver resection or liver transplantation as well as locoregional radio-ablative therapies, i.e., transarterial chemoembolization (TACE) and Yttrium-90 trans arterial radioembolization (Y90). At early diagnosis, most patients show a 5-year survival rate of about 59% compared to <15% in the advanced stage [4].

The current recommendation for the screening of HCC in high-risk populations, endorsed by the American Association for the Study of Liver Disease (AASLD) involves ultrasound (US) with or without alpha-feto protein (AFP) measurement at 6-month intervals [5]. Authors have argued that it is a simple, cost-effective, and noninvasive imaging modality available worldwide [6,7]. Nevertheless, the imaging by the US is operator dependent, adding to the changing growth pattern of tumors in their sonographic appearance from hypoechoic to isoechoic with a hypoechoic rim as tumor size increases. A meta-analysis by Tzartzeva et al. showed that in the asymptomatic patient, the US as imaging modality had a pooled sensitivity of 84% in detecting HCC [8]. However, its sensitivity drops to 47% in early-stage HCC. Presently, AFP is the most widely used biological marker for HCC (see Table 1). At a threshold of 20 ng/mL, it has a sensitivity of 63% and a specificity of 84% [5]. Other imaging modalities (multiphasic CT and MRI) have been shown to improve sensitivity from 66 to 82% and specificity to >90% for the detection of at least 1 cm in diameter hepatic nodules [9]. Albeit, the financial burden, adverse events (radiation), and lack of availability in specific geographical areas in development make CT/MRI imaging a non- cost-effective screening strategy.

Although a liver biopsy is not recommended for screening in patients with end-stage liver disease (ESLD), histological diagnosis may be indicated in non-cirrhotic patients with risk factors when their imaging is inconclusive for HCC. Liver biopsy is also required in patients who are not candidates for curative therapies before the start of systemic therapy [10]. The sensitivity of liver biopsy varies between 66–93% based on tumor size, operator experience, and needle size with specificity and a positive predictive value above 95% [11]. In complex cases, the accuracy of a liver biopsy is enhanced by using a combined panel of immunochemistry markers such as glypican 3 (GPC 3), heat shock protein 70 (HSP 70), glutamine synthetase (GS), and in some instances a panel of antibodies [1,12,13]. Complications from a percutaneous liver biopsy include pain, bleeding, gallbladder perforation, hemothorax, haemobilia, bile peritonitis, and pneumothorax. Severe bleeding within 2–4 h after biopsy occurred in less than 2% of patients [14], while mortality has been reported in 1 per 10,000 patients [11,15,16], and malignant needle tracking occurrence at a 2.7% overall rate, or 0.9% per year [17].

The molecular pathogenesis of HCC is complex and heterogenous, and conventional biopsy cannot always be done because of its invasiveness. Furthermore, the information obtained from a single biopsy may be inadequate and may not be entirely representative of the pathology due to tumor heterogenicity and sample bias. Sequential liver biopsies have been proposed to monitor tumor evolution, but besides being impractical, they add risk to the patient for adverse events. An alternative approach is to determine biomarkers in plasma that define liver pathology. A liver liquid biopsy may provide a genetic/metabolic profile for a liver cancerous lesion and the opportunity to follow growth evolution in terms of regression, or recurrence. In this review, we aimed to discuss the actual status of biomarkers for HCC, including their limitations and possible new areas for further research.

**Table 1:** Biomarkers and detection performance for HCC early detection. (Adapted and modified from Parikh et al., 2020) [18].

Biomarker Abbreviation	Biomarker Name	Early Detection Performance	AUC ROC for Early Detection
AFP	Alpha-fetoprotein	Sensitivity: 39–64% Specificity: 76–97%	0.75–0.82
AFP-L3	Lens culinaris agglutinin-reactive fraction of alpha-fetoprotein	Sensitivity: 49–62% Specificity: 90%	0.66–0.76
DCP	Des-gamma-carboxy prothrombin	Sensitivity: 34–40% Specificity: 81–98%	0.72
OPN	Osteopontin	Sensitivity: 49% Specificity: 72%	0.73
GPC-3	Glypican-3	Sensitivity: 55% Specificity: >95%	0.793
GP-73	Golgi protein 73	Sensitivity: 62–79% Specificity: 62–88%	Not Available

## 2. Concept

**Figure 1:**
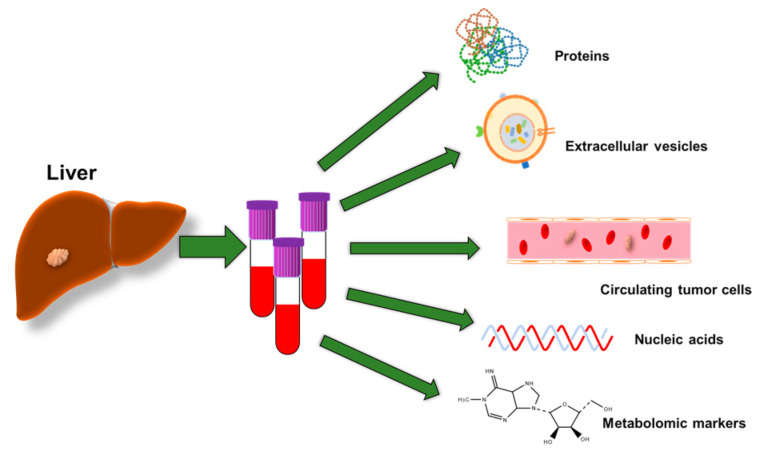
Potential biological markers for a liquid biopsy. Proteins, extracellular vesicles containing most of them RNA, circulating tumor cells, free DNA, and metabolomic markers are the main candidates for the development of a plasma liver construct for the early detection of HCC. Modified and adapted from Mocan et al., 2020 [20].

A liver liquid biopsy (LLBx) is a minimally invasive test that measures liver-originated by-products in plasma, i.e., proteins, circulating tumor cells, cell-free RNA, metabolomic markers, microRNA, and extracellular vesicles that precede morphological liver changes during the progression/regression of a benign or malignant liver disease process (Figure 1) [19,20]. It diminishes the financial burden and potential complications of tissue biopsies, but also can be performed sequentially defining disease evolution. This concept in a liver process has been validated for liver fibrosis, by correlating the grade of liver fibrosis with plasma liver enzymes in multiple clinical trials, i.e., Fib-4 [21,22,23,24,25,26,27]. The MELD score (modeling for end-stage liver disease) predicts mortality at 90 days in patients listed for liver transplantation, and it is the standard for liver graft allocation in the US and abroad [28,29,30]. Nevertheless, a reliable LLBx that predicts the development of HCC prior to its detection by imaging, remains to be determined and validated. We will review the candidacy of biomarkers for the early detection of HCC. 

## 3. Proteins

### 3.1. Alpha-Feto Protein (AFP)

AFP is a glycoprotein that resembles albumin and is produced by the fetal liver and yolk sac during embryonic development but is absent in adult life. AFP was identified in human fetal serum in 1956 [31], it has 590 amino acids organized in three domains that undergo conformational changes based upon temperature, pH, and osmolality. It was introduced in clinical practice as a biomarker in the 1990s [32], when it was described as a carrier for different ligands. Thus, AFP can alter and enhance signal transmission pathways, explaining its role in modulating cell growth in fetal life and oncogenesis [31,33,34]. Two isoforms of AFP have been described: native AFP (nAFP) and tumor AFP (tAFP). 

**Figure 2:**
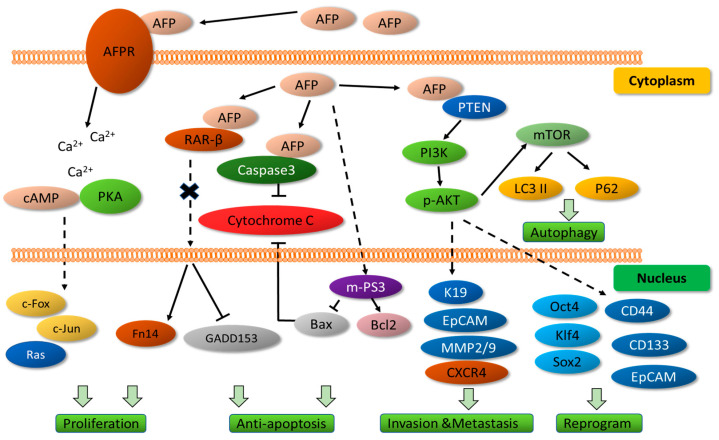
Molecular interactions of AFP. During fetal life, nAFP interacts at the membrane level with its receptor to promote cell proliferation and apoptosis inhibition. tAFP promotes cell division, organ invasion, and distal tumors. Modified and adapted from Li et al., 2020 [36].

nAFP is responsible for stimulating cell and tissue growth in the fetal stage. In adults, only a trace concentration (5–8 ng/mL) is available and helps to condition hematopoiesis and cell regeneration [31,33,34]. tAFP is synthesized by neoplastic hepatocyte signaling and, in turn, it drives the growth of cancerous cells, block apoptosis, and increase drug resistance (Figure 2) [35,36]. tAFP signal pathway follows the activation of the cAMP-PKA enhancing the expression of the c-jun, c-fos, and RAS oncogene promoting cell cycle transition from G1 to S phase and initiating angiogenesis [31,33,34]. In addition, by binding with phosphatase and tensin homolog (PTEN), tAFP causes the activation of the PI3K/P-AKT/mTOR pathway disabling the cell’s autophagy capacity [34,37,38]. Furthermore, the binding of tAFP to caspase 3 blocks the signal pathway from caspase 8 to 3, thereby inhibiting apoptosis. The anti-apoptotic effect is enhanced by its ligand capacity to both the hormone response element (HRE) and DNA damage-inducible protein 153/C/EBP homologous protein/DNA damage-inducible transcript 3 (GADD153/CHOP/DDIT3) [33,37]. tAFP has an effect on the expression of other proteins that play roles in metastasis, including keratin-19, EpCAM (epithelial cell adhesion molecule), matrix metalloproteinases 2 and 9 (MMP 2/9), and CXC chemokine receptor 4 (CXCR4) [36].

The threshold of tAFP varies depending on the prevalence of HCC in the population at risk [39]. Patients with non-tAFP HCC have better overall survival. They have smaller tumor sizes, superior liver function, a lower rate of tumor recurrence, and undergo liver transplantation at a higher rate [39,40,41]. Trevisani et al. using a cut-off of 20 ng/mL in a population with 5% HCC prevalence, found for tAFP a negative predictive value of 97% and a positive predictive value of 25% [39]. Patients with advanced HCC and tAFP < 400, had higher rates of partial or complete response to immune checkpoint inhibitors and a lower rate of disease progression compared to patients with tAFP > 400 ng/mL [40]. Patients with failure to normalize tAFP after liver transplantation for HCC, had a higher recurrence rate [41]. A retrospective study of 422 HCC patients who underwent liver transplantation confirmed that consistently high tAFP values (> 15 ng/dL) is an independent predictive factor for poor HCC outcome [7].

AFP is expressed by approximately 73% of patients with HCCs, making it eligible to be used as a marker for T-cell-mediated immunotherapy. AFP-directed vaccine for HCC is currently being studied in animals and clinical trials. Hanke et al. found that 62.5% of vaccinated mice with AFP-expressing plasmid-DNA rejected subcutaneous syngeneic AFP-expressing p815 tumors at a higher rate when compared to control animals (62.5% vs. 16.7%, respectively, *p* < 0.03), suggesting that AFP-specific DNA vaccination inhibit the growth of AFP-expressing tumors without affecting liver regeneration [42]. Other studies in mice models have shown that AFP may be used as a potential self-antigen to induce cytotoxic lymphocyte and CD4 (+) T cell-mediated regression of HCC-expressing AFP [43,44,45]. The creation of AFP-epitope optimization will enhance the activation of AFP-specific CD8 T cells which will lead to the cross-recognition of native AFP epitopes on HCC tumor cells generating an antitumor effect [46]. A phase I/II clinical trial reported the possibility of expanding AFP-specific CD8 T cell response in advanced HCC patients with high circulating levels of AFP after immunization with AFP peptide-pulsed autologous dendritic cells [47]. tAFP can be elevated in other embryonic tumors and in some benign liver conditions, that usually implicate liver regeneration. Therefore, high levels of tAFP are used in combination with imaging studies to confirm the diagnosis [48,49,50].

### 3.2. Des-Gamma-Carboxy Prothrombin (DCP)

This protein results from an acquired defect, in malignant cells of the posttranslational carboxylation in the prothrombin precursors. It is also known as prothrombin induced by vitamin K absence-II (PIVKA-II). Secretion of DCP is independent of tAFP, hence it is described as a useful tool for HCC surveillance [51]. However, its use remains controversial. Studies have shown that DCP presented the highest diagnostic value for discriminating HCC vs. control with an adjusted AUC of 0.82 (95% CI 0.64–0.80), higher than the traditional tAFP (AUC = 0.72, 95% CI 0.70–0.82, *p* = 0.045) [52]. Additionally, DCP was shown to be significantly better than tAFP or AFP-L3 in differentiating HCC from cirrhosis with a sensitivity of 86% and specificity of 93% [53]. DCP has also demonstrated usefulness in tumor progression and prognosis. In a prospective study on 685 HCC patients, AFP and DCP showed similar discrimination (55.8 and 54.2%, respectively) while AFP-L3 was lower (34.1%) [54]. In addition, HCC AFP-negative patients (approximately 30%) appear to be DCP-positive and these patients usually display malignant lesions possessing a distinct margin, few nodules, and a larger size of >3 cm with moderate to poor differentiation [55]. Furthermore, a higher level of DCP is associated with intrahepatic metastasis, hepatic tumor vein thrombosis, and portal vein tumor invasion, driving a correlation between HCC stages and survival [56]. Studies have suggested that DCP may play a role in the release of angiogenesis molecules in HCC, and vascular endothelial cells [6,56].

The half-life in the plasma of DCP is shorter than AFP (40–72 h vs. 5–7 days, respectively). DCP levels may be affected in intrahepatic cholestasis, prolonged obstructive jaundice, vitamin K deficiency, patients taking warfarin, and therapy with antibiotics [57]. Since DCP is produced by vitamin K shortage, extrinsic supplementation of the vitamin may decrease the serum levels of DCP and even reduce the HCC burden. In the nude mice bearing HCC, vitamin K2 (2–40 uM) significantly decreased DCP production and inhibited HCC growth, invasion, and migration of tumor cells [58]. Using PRF/PLC/5/hepG2 cells, Vitamin K2 effects were confirmed to have no effect on hepatocyte viability [59]. Given the potential promise of DCP, it is currently approved by the FDA for use in the determination of HCC [5,56]. Japan has recommended the combined use of DCP and AFP [6]. Nevertheless, Choi et al. disagree with the addition of DCP to AFP for increasing HCC detection [53]. The AASLD has no recommendations on DCP for surveillance and diagnosis of HCC [6,56].

### 3.3. AFP-L3 (Lens Culinaris Agglutinin-Reactive Fraction of Alpha-Fetoprotein)

AFP-L3 is one of the fractions of the AFP separated by its affinity to the lectin *Lens culinaris* agglutinin (LCA) [60]. Elevated levels of AFP-L3 predicted HCC even in the absence of elevated tAFP [61]. AFP-L3 has a sensitivity of 56% with a specificity of 96% and can be detected in the serum of about 35% of patients with small HCC (<2 cm) [58]. AFP-L3 specificity is advantageous in being able to decipher HCC from another benign liver disease in the presence of elevated AFP [59]. However, due to its low sensitivity, it does not seem suitable for the first-line screening of early HCC. A cut-off value of 5% was associated with lower overall survival, and a higher risk of HCC recurrence, even in those with AFP value of less than 20 ng/mL [62,63]. Just as DCP, AFP-L3 is also associated with intrahepatic metastasis and portal vein invasion which explains its usefulness in the monitoring of treatment response and HCC recurrence [64,65]. With continuous efforts being made to improve the performance of AFP-L3 by combining it with other biomarkers, some method like the GALAD score which entails age, sex, AFP, AFP-L3, and DCP has shown significant improvement in diagnostic performance for early-stage HCC.

### 3.4. Glypican-3 (GPC-3)

GPC-3 protein belongs to the family of heparin sulfate proteoglycans and is anchored to the plasma membrane by a glycosylphosphatidylinositol ligand. It regulates cell proliferation and survival during embryonic development and plays a pivotal role as a tumor suppressor. The Glypican family comprises six members (GPC1-GPC6), all of which have a cysteine-rich repeat domain at similar positions. GPC3 is abundantly expressed in the placenta and fetal tissues, i.e., liver, lungs, and kidneys but its expression is significantly reduced in adult organs. Although GPC-3 is downregulated in breast, ovarian, and lung cancers, it is upregulated in HCC [66,67]. GPC mRNA is hardly detectable in non-cancerous adult liver but over-expressed in HCC tissue [68]. The GPC-3 released by HCC cells is detected as the serum GPC-3 (sGPC3) and is measured using the enzyme-linked immunosorbent assay (ELISA). sGPC-3 levels in 25 healthy volunteers showed significantly lower levels when compared to 115 HCC patients who underwent curative partial hepatectomy (110.12 vs. 405.16 pg/mL, respectively; 185.52 pg/mL was set as the upper limit of normality) [69].

GPC-3 can be detected in HCC patients that are AFP and DCP seronegative (50 and 33%, respectively). Its expression is independent of tumor size and can exhibit a sensitivity of 56% in patients with early-stage tumors (<3 cm) [70]. Newly diagnosed HCC patients (n = 449) with serum GPC-3 > 150 pg/mL had lower overall survival (16; 95%CI: 13–24 months) than those with GPC-3 ≤ 150 pg/mL (36; 95%CI: 30–56 months, *p* < 0.001) [69]. Higher expression of GPC-3 in HCC cells has been found to correlate to poorer prognosis for those with curative hepatectomy [71,72]. GPC-3 also has some role to play in immunotherapy. Codrituzumab/GC33/RO5137382 (GC33) is a humanized monoclonal antibody that binds to human GPC-3 expressing HCC cell line with an in vivo cytotoxic activity against tumor cells. In a phase-1 trial, 20 patients were enrolled and treated with GC33. Although the maximum tolerated dose was not reached, only a few non-severe adverse events such as constipation, hyponatremia, headache, and fatigue were documented [73].

### 3.5. Osteopontin (OPN)

This is an integrin-binding glycol-phosphoprotein expressed in epithelial cells and is believed to be involved in the regulation of migration, invasion, and metastasis of tumor cells. OPN expressions have been reported to be significantly higher in HCV-related HCC patients compared to healthy individuals [74]. OPN levels have also been correlated to advanced HCC stage, vascular invasion, lymph node metastasis, and poor prognosis. In a meta-analysis study, pooled sensitivity, specificity, and diagnostic odds ratio for OPN vs. tAFP were 0.813 vs. 0.639 (95% CI: 0.671–0.902 vs. 0.538–0.729), 0.874 vs. 0.959 (95% CI: 0.778–0.932 vs. 0.909–0.982), and 30.047 vs. 41.518 (95% CI: 8.845–102.067 vs. 13.688–125.929), respectively. In addition, pooled sensitivity, specificity, and diagnostic odds ratio for OPN+tAFP were 0.856 (95% CI: 0.760–0.918), 0.738 (95% CI: 0.630–0.823), and 16.718 (95% CI: 7.950–35.156), respectively [75]. Another meta-analysis comparing OPN and AFP in the evaluation of HCC reported a similar trend [76].

### 3.6. Survivin and Smac-Diablo

#### 3.6.1. Survivin

Survivin, an oncofetal protein is a member of the inhibitor of apoptosis protein (IAP) family and chromosomal passenger complex. It is a pivotal protein in many molecular pathways in the cell [77,78]. Primarily, it inhibits apoptosis, drives cell proliferation, and promotes angiogenesis [77,79]. The expression of survivin is upregulated in HCC and other forms of cancer when compared to normal tissues and its high expression is associated with carcinogenesis [77,79]. Interestingly, the presence of survivin can be detected from the extracellular fluid (ECF) of cancer patients and accumulating data indicates that measurement of survivin level in the ECF could be a potent diagnostic biomarker for early detection, diagnosis, and prognosis of various types of cancer and foretell clinical outcome to various anti-neoplastic therapies [77,80]. Assessment of survivin level in the urine of patients with bladder cancer has been shown to be a simple, but effective diagnostic technique for detecting new or recurrent bladder tumors [80,81]. In addition, circulating breast cancer cells expressing survivin mRNA in peripheral blood, were present in more than 50% of breast cancer patients and absent in healthy subjects, correlating its presence with metastasis and recurrent breast tumors [80,82]. The evaluation of serum levels of survivin in cancer patients could be an effective diagnostic biomarker for early detection of tumors and disease progression [83,84]. Intriguingly, preliminary data from our group, under approved IRB protocols, has demonstrated an increased level of survivin in the plasma samples of MASH-related HCC patients compared to MASH patients and normal subjects, which correlated positively with the clinicopathological parameters of the patients. The plasma level of survivin in these patients was able to effectively discriminate between MASH patients and those that have developed cancers. Taken together, plasma survivin is a putative effective biomarker for early detection of MASH-related HCC and perhaps, as a screening tool in high-risk communities.

#### 3.6.2. Smac/DIABLO

The second mitochondria-derived activator of caspase/direct inhibitor of apoptosis-binding protein with low isoelectric point (pl) (SMAC/DIABLO), is a proapoptotic protein that is released from the mitochondria into the cytosol following various apoptotic stimuli. In the cytosol, SMAC binds and neutralizes inhibitors of apoptosis proteins (IAPs), promoting the activation of caspases and apoptosis [79,85]. SMAC is down-regulated in HCC tumor tissues in comparison to normal tissues, thus aiding hepatocarcinogenesis via decreased apoptosis [79,86,87]. In ovarian cancer patients, despite SMAC not being a secretory molecule, it can be detected in the serum and its circulating level could be a useful diagnostic and prognostic biomarker in cancer. Furthermore, the circulating level of SMAC was downregulated in patients with bladder cancer when compared to control subjects. The decreased serum level of SMAC correlated negatively with both the progressive cancer stage and early recurrence of tumors in these patients, indicating that serum levels of SMAC may be a prognostic biomarker for bladder cancer [88]. Dobrzycka et al. showed in patients with serous ovarian cancer, a positive correlation between decreased SMAC serum levels and poor prognosis, predicting their clinical outcome [89]. In malignancy, reduced apoptosis occurs, with the low release of SMAC in circulation [88,89]. Our data, under approved IRB protocols, revealed an increased circulating level of SMAC in the plasma samples from MASH patients when compared to normal subjects. Nevertheless, SMAC levels were not significantly different from patients with MASH-related HCC. SMAC is also identified as a potential biomarker for MASH progression to HCC.

## 4. Liver Apoptotic Activity-Cytokaretin-18 Fragment

It is well-documented that elevated hepatocyte apoptosis is a prominent feature of MASH. During apoptosis, activation of the executioner caspases, especially caspases 3 and 7 results in the cleaving of certain substrates in the cell, including cytokeratin-18 (CK-18), which is the major intermediate filament in the liver. The resulting caspase-cleaved cytokeratin -18 fragments circulate in the peripheral blood of MASH patients and its plasma/serum level has been correlated with the severity of MASH [90,91]. Feldstein et al. reported that CK-18 fragments are highly elevated in the blood of MASH patients and serve as an independent predictor of MASH, detecting the presence of MASH with a specificity >90%, and a negative predictive value of 80% [90]. Our preliminary data, under approved IRB protocols, concurred with the findings where caspase-cleaved CK-18 fragments plasma levels were heightened in MASH patients in comparison to healthy controls, but not statistically different from those with MASH-related HCC. This finding further establishes the circulating level of caspase-cleaved CK-18 fragments as a valuable diagnostic biomarker for MASH progression.

## 5. Cell Autophagy Activity

Autophagy is an evolutionarily conserved mechanism, that involves a lysosome-mediated intracellular degradation and recycling pathway [92]. It has roles in a variety of pathophysiological processes, mainly where there is a high protein turnover. Cell autophagy regulates metabolism and cell protein renewal [93,94]. During aging, autophagy actively participates in the elimination of toxic proteins and defective organelles [95]. Autophagy has a dynamic role in both the promotion and prevention of cancer through cell survival or tumor initiation blockage [96,97,98]. Autophagy is affected in HCC pathogenesis and possesses a potential role in tumorigenesis and treatment resistance [99,100]. Microtubule-associated protein 1 light chain 3 (LC3) and Beclin-1 are autophagy marker genes associated with the pathogenesis of liver diseases [101,102], and are reported as having controversial roles in the pathogenesis of HCC [103,104,105]. LC3 has been associated with vascular invasion and poor tumor survival [102,103,106], insinuating autophagy-related proteins may predict tumor survival in patients with combined hepato-cholangio carcinoma [107]. Furthermore, LC3B has been found to be positively correlated with cytokeratin 19-labeled ductular reaction in the development of cirrhosis [108], and vascular invasion and lymph node metastasis in HCC [109]. LC3A has also been reported as a marker for HCC progression [110]. UNC51-like kinase 1 (ULK1), a mammalian serine/threonine protein kinase that plays a crucial role in the initiation of autophagy, was also identified as a novel prognostic biomarker for HCC [111]. Plasma extracellular vesicles expressing LC3B have been reported as a diagnostic marker to predict HCC [112]. The absence of LC3, hypoalbuminemia, high alanine aminotransferase (ALT), and major liver resection were associated with HCC mortality [113].

## 6. DNA/RNA

### 6.1. Circulating Tumor Cells (CTCs)

CTCs are shed cells from a malignant nest in proliferation providing the foundation for metastatic disease. Since their discovery in 1869, researchers have shown that CTCs can undergo an epithelial–mesenchymal transition (EMT), resembling stem cell-like characteristics that enable cell self-renewal and differentiation [114]. Cell search defined CTC as a circulating nucleated cell larger than 4 µm, expressing epithelial proteins (EpCAM, cytokeratin’s 8, 18, or 19) but negative for leucocyte-specific antigen CD45 [115]. The cell search system (FDA-only validated assay) is used in detecting CTC in HCC. It is important to note that the earlier the cancer stage, the lower the number of circulatory CTCs. Also, when this marker is in the circulatory system, it undergoes apoptosis because of loss of adhesion to the extracellular matrix, the hemodynamic shear forces, target drugs, and attack of the body’s immune system. Therefore, less than 0.01% of CTCs released into circulation survive to produce metastasis [116]. This can limit the detection of an early cancer diagnosis. The half-life of this marker is 1–2.4 h and the detection in the patient’s blood after months to years of primary tumor resection could indicate tumor recurrence or metastasis [45].

CTCs are believed to be determinants of metastasis and recurrence in cancers, but CTCs may also play a role in determining disease prognosis. There have been multiple parameters in defining a CTC-positive cell assay. Some studies defined it as “≥1 CTC” while others “≥2 CTC”, or “≥5 CTC [2]. In HCC patients, CTC-positive is associated with lower overall survival and disease-free survival and poor clinical characteristics, and larger CTC numbers correlate to poorer prognosis [2]. CTCs may also predict tumor recurrence. A total of 123 HCC patients had their blood samples tested (CTCs+ in 66.67%) prior to liver resection and one month thereafter [117]. Patients with CTC’s > 2 developed HCC recurrence earlier when compared to patients with CT < 2 (*p* < 0.001). Another study showed that CTC count ≥16 and mesenchymal-CTC (M-CTC) percentage ≥2% prior to resection were significantly associated with early recurrence, multi-intrahepatic recurrence, and lung metastasis [118]. In 49 patients high CTC/Treg levels showed a significantly higher risk of developing postoperative HCC recurrence than those with low CTC/Treg levels (66.7% vs. 10.3%, *p*  <  0.001) [119]. Authors concluded that the number of *EpCAM* ^mRNA+^ CTCs and Treg/CD4^+^ cells showed a significant correlation as prognostic factors of postoperative HCC recurrence.

### 6.2. Circulating Tumor DNA (Ctdna) and Cell-Free DNA (Cfdna)

CtDNA is released from primary or metastatic tumors while cfDNA is a double-stranded DNA, released from dying nonmalignant host cells and lymphocytes. Thus, ctDNA represents only a fraction of the total cfDNA with DNA dilutions from neighboring non-cancerous cells. The detection of tumor-specific mutations on circulating cfDNA indicates the presence of ctDNA [120]. ctDNA modal size is 167 bp, shorter than cfDNA; however, a 90–150 bp range is sometimes used in the selection of fragments to improve detection [121]. ctDNA can be difficult to detect due to dilution with cfDNA, and short half-life (16 min–2.5 h). Some characteristics possessed by ctDNA, i.e., cancer-derived viral sequences, single nucleotide mutations, and methylation changes can be used to differentiate it from circulating cfDNA [122,123]. Quantitative ctDNA changes reflect disease activities, response to treatment, or tumor recurrence. Previous studies showed that CfDNA differentiates HCC patients from healthy control with a sensitivity of 90.2% and specificity of 90.3% using a cut-off value of 18.2 ng/mL, and its levels were positively associated with tumor size (*p* = 0.012) [124]. Furthermore, elevated cfDNA was significantly associated with intrahepatic spreading or vascular invasion (*p* = 0.035) and overall survival (*p* = 0.071). At a higher cut-off level (73 ng/mL), ctDNA discriminated HCC patients from HCV carriers and controls (sensitivity of 69.2% and specificity of 93.3%), and its levels were associated with tumor differentiation and size but not with gender, TNM stage or levels of other proteins (tAFP and DCP) [125]. Ren et al. showed that the cfDNA levels were significantly associated with HCC size (*p* = 0.008) and TNM stage (*p* = 0.040), and negatively associated with disease-free survival (DFS at 3 years, *p* = 0.017) and overall survival (OS, *p* = 0.001) [126]. A combined measurement of cfDNA with tAFP may increase the HCC detection rate [127]. A case–control study by Lewin et al. used DNA methylation markers (mSept9) in 60 HCC patients and 103 cirrhotic patients without HCC; 46/60 HCC and 37/103 cirrhotic patients tested positive to mSept9 with sensitivities of 76.7% and 64.1%, respectively. All patients also had AFP results which showed a sensitivity of 36% and specificity of 95% using a cuff-off value of >20 ng/mL; however, when combined with a methylated biomarker panel, sensitivity improved to 68% with specificity at 97% [128].

### 6.3. Cell-Free Messenger RNA

Cell-free messenger RNAs were initially thought to be of low diagnostic value in HCC, because of their low quantity and ease of degradation by blood ribonucleases. Nonetheless, recent studies have shown they are incorporated into exosomes, micro-vesicles, and multi-vesicles (see below).

### 6.4. Non-Coding RNAs (ncRNAs)

Messenger RNAs that do not code for protein are transcribed into ncRNAs as long ncRNA (LncRNA) or short ncRNA (SncRNA). Metastasis-associated Lung Adenocarcinoma Transcript 1 (MALAT1) and Sprouty Receptor Tyrosine Kinase Signaling Antagonist 4-Intronic Transcript 1 (SPRY4-1T1) LncRNAs (with an average length of 21–25 nucleotides) are increased in the serum of HCC patients and are found to correlate with the grade of HCC differentiation [129]. Other LncRNA (XLOC014172, LINC00152, and RP11-160H22.5) have been found to be able to distinguish HCC from chronic hepatitis, cirrhosis, or controls (normal liver) [130].

An example of a SncRNA is the miRNAs (longer than 200 nucleotides), which are small, dysregulated RNAs (19–25 nt) protected from endogenous RNA activity. miRNA regulates several cell processes like development, differentiation, metabolism, and cell death, and has acted as a useful biomarker in HCC detection. Various characteristics of miRNA have been described and these include resisting apoptosis and immune destruction, promotion of inflammation and gene mutation and instability, and playing a role in angiogenesis [131,132,133,134,135,136,137]. The authors studied miRNAs (miR-21, miR-141, and miR-122) and found elevated levels in lymphoma, prostate, and liver injury [138]. miR-221 has been found to be expressed by tumor cells like breast, colorectal, and glioblastoma with a report showing its ability to enhance tumor progression and shorten the mean time to death in a mouse model of liver cancer. Li et al., however, reported elevated serum levels and expression of miR-221 in HCC patients, correlating higher expressions with tumor size and stage compared to those with low expression [139]. Oncofetal miRNA, miR-500 was abundantly expressed in the serum of HCC patients and tended to decrease following surgical treatment [127]. Shen et al. compared miRNAs by ethnicity and discovered higher levels in Asians compared to non-Asians, with some having a different expression pattern [140]. MiR-125b-5p was found to be associated with HCCs for both Turks and Chinese people; however, its expression was downregulated in Chinese patients and upregulated in Turks. These ethnic differences were believed to be due to heterogenous pathogenesis, diet, environmental exposures, and lifestyles. The diagnostic value of miRNA could be limited when screened alone. miR-21 has a sensitivity and specificity of 60% and 83%, respectively, when screened alone but when combined with AFP improved to a sensitivity and specificity of 81% and 77%, respectively [141].

### 6.5. Epigenetic Changes

Genome-wide studies, from the use of next-generation DNA sequencing, have explored a multitude of genetic and epigenetic aberrations associated with the process of liver carcinogenesis [142]. As the liver constantly adapts to highly variable environmental conditions and is subjected to constant repair and regeneration, undesirable changes in the liver epigenome drive uncontrolled cell proliferation, invasion, and metastasis that leads to HCC progression [143,144]. Major epigenetic modifications include DNA methylation, histone modifications, and altered expression of non-coding RNAs [145]. The prognostic role of one of the most widely studied epigenetic modifications, DNA methylation, has been reported in HCC [146,147,148,149]. In patients with HCC after hepatectomy, serum insulin-like growth factor-binding protein 7 (IGFBP7) methylation status possesses potential prognostic value for recurrence and survival [150]. Plasma hypomethylation of long interspersed nucleotide elements-1 (LINE-1) was found to be associated with worse survival in patients with HCC [151]. Evidence suggests that hypo-methylation of promoters of CTCFL (CCCTC-binding factor-like), a member of the cancer-testis antigen family, can be used as a noninvasive biomarker to monitor HCC prognosis [152]. Methylation of a tumor suppressor gene, Septin 9 (SEPT9), can serve as a promising circulating epigenetic biomarker for HCC diagnosis [153].

A predictive model of circulating tumor DNA carrying cancer-specific genetic and epigenetic aberrations was constructed from patients’ cell-free samples with HCC vs. normal controls, and the model showed high diagnostic specificity and sensitivity for HCC [154]. In addition, another study has demonstrated the diagnostic value of serum G-protein-coupled bile acid receptor Gpbar1 (TGR5) methylation in HCC and chronic hepatitis B patients [155]. A multicenter, case–control study developed a blood-based biomarker panel of methylated DNA and protein markers with high sensitivity for early-stage HCC detection to increase treatment opportunities and reduce mortality (NCT03628651) [156,157]. Oncoguard Liver™ uses an algorithm based on sex, age, and alpha-fetoprotein (AFP) along with methylated homeobox A1 (HOXA1), empty spiracle homeobox 1 (EMX1), and TSPY-like 5 (TSPYL5) to improve early detection of HCC [156,157]. HelioLiver Test™, a multi-analyte blood test, combines cell-free DNA methylation patterns, clinical variables, and protein tumor markers for HCC detection (NCT05059665) [158]. The study measured cell-free DNA methylation levels in 28 genes having 77 CpG sites. Hence, future studies are warranted to explore aberrant epigenetic modifications as promising noninvasive biomarkers to sensitively detect early HCC development.

## 7. Extra Cellular Vesicles (EV)

Exocytosis of cytoplasmic synthetized vesicles containing a variety of biological compounds, i.e., mRNAs, hybrid miRNAs, proteins, and lipids, is part of cell physiology in health and disease. Based on their biogenesis and size they are designated as exo/ectosomes, micro-vesicles, and apoptotic bodies. Exosomes are small membrane-enclosed vesicles (typically below 30–120 nm in diameter) generated by the inward budding of the membrane (endocytosis) while ectosomes are formed by the outward blebbing of the plasma membrane released by proteolytic cleavage (exocytosis) into the extracellular compartment. While micro-vesicles are bigger in size, ranging from 100 nm to 1 μm, and are derived from the budding of the membrane, apoptotic bodies have the largest diameter (1 to 5 μm) and are formed by compartmentalization of the cell after nuclear material dissolution (programmed cell death) [159].

Exosomes are heterogenous and contain cell-specific proteins and nucleic acids (mRNAs, miRNAs, and ncRNAs), reaching up to 3 million units/µL in peripheral blood [159]. In 2012, the Exocarta database described 11,261 proteins, 2375 mRNAs, and 764 miRNAs associated with exosomes [160], some of them used by cancer cells to reprogram adjacent healthy cells [161]. In chronic liver disease, which involves systemic inflammation, an increased number of EVs derived from inflammatory cells (CD8 and CD4^+^ cells) have been reported. They have been found to be increased in chronic HCV and alcohol-induced but decreased in MASH [162,163,164]. Alcohol- or HCV-induced cirrhosis has been shown to have elevated levels of leuko-endothelial (CD31^+^/41^−^), lymphocyte (CD4^+^), pan-leukocyte (CD11a^+^), erythrocyte (CD235a^+^), and cytokeratin-18 (CK18) positive micro-vesicles [165]. To overcome a low specificity barrier that comes from myeloid cell derived EVs released following a variety of inflammatory conditions, some groups have sought to screen for the levels of specific liver progenitor cell markers, i.e., vanin-1 and cytokeratin-18 [166].

## 8. Cell Oxi-Redox: The Glutathione System

The tri-peptide glutathione (glutamate–cysteine–glycine=GSH) and its oxidized disulfide counterpart (GSSG) form a redox couple which is part of the line of defense of many cell types against oxidative damage by reactive oxygen species. In rats, the small pool of plasma GSH/GSSG is derived mostly from the liver because the amount of GSH released in plasma + bile is equal to the rate of GSH synthesis in the liver [167]. It had been proposed to use the labeling kinetics of plasma GSH from 2H-enriched body water as a proxy for the kinetics of labeling of liver GSH. Interestingly, we showed that the production of 2H-enriched GSH by human livers is significantly higher in controls than in cirrhotics or patients with HCC [168]. Indeed, glutathione sp. was one of the biological markers able to differentiate controls from patients with ESLD and HCC.

## 9. Metabolomics

The metabolic profile from measuring low molecular metabolites in biological samples (e.g., blood, urine, bile, ascites, tissue, etc.) may provide signatures of health and specific disease processes. It has offered a unique approach to identifying unique biomarkers elucidating biochemical pathways in human malignancy. The liver is a metabolic organ that processes proteins, lipids, and carbohydrates after absorption from the intestine. Nevertheless, the study of metabolomic profiles in chronic liver disease is in its infancy. The most frequently reported metabolites in chronic liver disease are *bile acids*. Higher serum bile acids have been found in patients with cirrhotic HCC patients compared to healthy individuals [127,169,170,171]. Nine *lyso-phosphatidylcholines* (LPCs, LPC C16 : 0, LPC C18 : 0, and LPC C18 : 2; LPC C18 : 0 and LPC C16 : 0) have been reported to be decreased in the serum of HCC patients [172]. Differences in LPC’s may be explained by the overexpression of lysophospha-tidylcholine acyltransferase 1 (LPCAT1), which converts LPC C16 : 0 to phosphatidylcholine 18 : 1 [172,173]. Petterson et al. showed that lignoceric acid (24:0) and nervonic acid (24:1) were absent in plasma from patients with HCC [174]. A trend was reported towards increased urinary levels of free carnitine in HCC patients when compared with healthy control or cirrhotics [170]. However, renal impairment was not considered in these studies. Serum carnitine levels in HCC, when compared to healthy adults, exhibit a specific pattern that includes increased free carnitine levels, decreased short- to medium-chain acylcarnitine, and increased long-chain acylcarnitine C18:1 and C18:2. Others have shown different carnitine profile levels in cirrhosis from varied etiologies. Krahenbuhl et al. reported an increase in plasma long-chain acylcarnitine in viral hepatitis cirrhosis compared to alcohol-induced liver cirrhosis that showed elevated levels of both the long- and short-chain acylcarnitines [175].

Metabolic signatures of HCC have been developed in VX2 rabbit and HCV ferret models for HCC [176,177,178]. In human plasma, glutathione sp., glucose, lactate, and glycerol discriminate patients with ESLD from controls and patients with HCC [168]. Other amino acids and lipids may play a major role in selecting a more sensitive and specific signature. Our preliminary work in the MASH and MASH-HCC mouse models showed promising results.

## 10. Glycosylated Protein Markers

Recent data have identified aberrant glycosylation of proteins in malignant cells as one of the principal hallmarks of cancer initiation and progression [179]. In HCC, there is an increased level of fucosylation, sialylation, and branching structures in serum among HCC patients with and without advanced fibrosis [179,180,181,182], driving aberrant protein’s glycosylation as a biomarker for HCC [179]. Current tumor-related glycosylated markers for HCC include:

Fucosylated Kininogen and alpha-1 antitrypsin (AAT). Fucosylated kininogen has been shown to be a biomarker for early detection of HCC. Albeit, it is not very efficient as a standalone marker, but when combined with AFP and other clinical signatures, it provides a robust performance with an AUC ROC of 0.97 [18]. Wang and his co-workers developed the Doylestown algorithm (a logistic regression algorithm that incorporates AFP, age, sex, alkaline phosphatase, and alanine aminotransferase) for early detection of HCC [183]. Their study showed that for HCC patients with AFP < 10 ng/mL, the Doylestown did not improve the detection rate of HCC (AUROC of 0.6417 for the Doylestown algorithm and 0.6313 for the AFP alone) compared to those with high AFP (10–100,000 ng/mL), where AUROC was 0.579 for AFP alone and 0.700 for the Doylestown algorithm. Nevertheless, their later report showed that the addition of lectin reactive low molecular weight kininogen to the algorithm increased performance and degree of sensitivity and specificity in the early detection of HCC, especially in those with AFP levels of <20 ng/mL [184]. Furthermore, patients with cirrhosis and HCC had higher levels of both AAT and kininogen than those with cirrhosis alone, and combining kininogen and AAT with AFP and Golgi protein 73 resulted in an increased sensitivity of 95%, a specificity of 70%, and AUROC of 0.94 for the detection of HCC [185].

Ceruloplasmin. Most of the serum markers of HCC are produced by the liver, including ceruloplasmin. The ratio of fucosylated glycopeptides from ceruloplasmin (CERU) has also been found to be elevated in patients with alcoholic hepatitis, HBV, and HCV [186]. Lin et al. found a significant elevation of fucosylated ceruloplasmin in HCC (35 cirrhotic vs. 27 early-stage MASH-HCC, *p* = 0.0486), raising the possibility of a fucosylation ratio as a potential marker for early detection of MASH-related HCC [186]. Casaril et al. reported on 159 patients (110 cirrhosis and 49 HCC), where they found in serum a significantly higher level of copper and CERU compared to their cirrhotic counterparts [187].

Glycosylated Haptoglobin (Hp). Haptoglobin (Hp), an acute-phase response protein secreted by the liver, functions to modulate renal iron loading and prevent kidney damage by releasing iron [188]. Previous studies have shown that serum haptoglobin (Hp), containing four N-glycosylation sites is a reporter molecule for aberrant glycosylation in tumorigenesis, including hepatocarcinogenesis and could serve as a highly specific biomarker for HCC [189]. Fucosylated as well as sialylated glycan structures of serum Hp have been shown to be significantly elevated in patients with HCC when compared to cirrhotics without tumors [188,189], Zhu et al. demonstrated a significant elevation of five N-glycopeptides at sites N184 and N241 during the progression of MASH to HCC (*p* < 0.05), with an AUC of 0.733 and 0.775, respectively. When combined with AFP, the two panels improved the sensitivity of early MASH-HCC detection from 59% (AFP alone) to 73%. The heightened bi-fucosylation level of Hp effectively discriminated early-stage HCC patients from cirrhosis and thus serves as a potential biomarker for early detection as well as for predicting HCC in cirrhotic patients [190]. Ang et al. reported significantly higher serum concentrations of Hp in HCC patients, hypothesizing an improved diagnostic accuracy of HCC with the combined use of Hp and AFP [191].

Other glycosylated markers for HCC include fucosylated proteins such as hemopexin, fetuin-A, serum paraoxonase 1, and histidine-rich glycoprotein. Increase levels of these markers have been observed in the serum of HCC patients via the use of techniques such as direct glycan sequencing or lectin-based procedures [18,179].

## 11. Methods for Identification of HCC Biomarkers

According to the World Health Organization, a biomarker is defined as: “Any substance, structure, or process that can be measured in the body or its products and influence or predict the incidence of outcome or disease [192,193].” Understandingly, most of the biomarkers discussed above are measured in the body fluids (e.g., plasma, serum, and urine) of HCC patients to serve as signatures that detect or predict cancer initiation and progression. Historically, the various methods used for the detection of these biomarkers in extracellular fluids include enzyme-linked immunosorbent assay (ELISA), immunoassays, microarrays, chemiluminescence, and immunoblotting techniques [179,194]. Furthermore, most recently, lectin-based assays and mass-spectrometry have emerged as very useful diagnostic techniques for biomarker evaluation. Lubman and co-workers have shown that mass-spectrometry is a valuable diagnostic tool, especially for glycosylated markers in HCC detection and diagnosis [179,189,190].

## 12. Future Directions

Integrating driving proteins, DNA-based, and metabolites in the progression of MASH to ESLD and HCC as biomarkers, is a promising undertaking for the modeling of algorithms in the screening for early-stage HCC detection in high-risk populations. Early-stage HCC detection will improve overall survival, decrease financial burden, and improve individual quality of life. Ideally, the LLBx construct will be performed within 24 h, as a routine test with a positive and negative predictive value over 90%. It also would not only discriminate patients with early HCC, but also treatment response and early recurrence.

## Abbreviations

AASLD: American Association for the Study of Liver Disease; AFP: alpha-fetoprotein; AFP-L3: *Lens culinaris* agglutinin-reactive fraction of alpha-fetoprotein; AKT: Protein Kinase B; ALT: alanine aminotransferase; AUC: area under the curve; cAMP: cyclic adenosine monophosphate; cfDNA: cell-free DNA; CHOP: C/EBP homologous protein; CTCFL: CCCTC-binding factor like; ctDNA: circulating tumor DNA; CTCs: circulating tumor cells; CT: computed tomography; CXCR4: CXC chemokine receptor 4; DCP: Des-gamma-carboxy prothrombin; DDIT3: DNA damage-inducible transcript 3; DFS: disease-free survival; DNA: deoxyribonucleic acid; EpCAM: epithelial cell adhesion molecule; EMT: epithelial–mesenchymal transition; EMX1: empty spiracle homeobox 1; ESLD: end-stage liver disease; ELISA: enzyme-linked immunosorbent assay; ECF: extracellular fluids; EV: extracellular vesicles; Fib-4: fibrosis index based on 4 factors; GADD153: DNA damage-inducible protein 153; GALAD score: (gender, age, AFP-L3, AFP, DCP) model score; GC33: Codrituzumab/GC33/RO5137382; GPC 3: Glypican 3; GSH: reduced glutathione; GSSG: oxidized glutathione; GS: glutamine synthetase; HBV: Hepatitis B virus; HCV: Hepatitis C virus; HCC: hepatocellular carcinoma; HSP 70: heat shock protein 70; HOXA: Homeobox A, HRE: hormone response element; ICC: cholangiocarcinoma; IAP: inhibitor of apoptosis protein; IGFBP7: insulin-like growth factor-binding protein 7; LC3: microtubule-associated protein 1 light chain 3; LC3A: microtubule- associated protein 1 light chain 3 alpha; LC3B: microtubule-associated protein 1 light chain 3 beta; LINE-1: long interspersed nucleotide elements-1; LncRNA: long non-coding RNA; LPCs: lyso-phosphatidylcholines; LPCAT1: lysophospha-tidylcholine acyltransferase 1; MELD: Model for End-stage Liver Disease; MALAT1: Metastasis-associated Lung Adenocarcinoma Transcript 1; mTOR: mammalian target of rapamycin; MASH: metabolic dysfunction-associated steatohepatitis; MASLD: metabolic dysfunction-associated steatotic liver disease; MMP 2/9: matrix metalloproteinases 2 and 9; mRNA: messenger ribonucleic acid; miRNAs: MicroRNAs; MRI: magnetic resonance imaging; nAFP: native alpha-fetoprotein; ncRNAs: non-coding RNAs; OPN: osteopontin; PKA: protein kinase A; PI3K: phosphatidylinositol 3-kinase; PTEN: phosphatase and tensin homolog; ROI: reactive oxygen intermediates; RNA: ribonucleic acid; SMAC/DIABLO: second mitochondrial activator of caspases/direct inhibitor of apoptosis-binding protein with low isoelectric point (pl); SncRNA: short non-coding RNA; SEPT9: Septin 9; SPRY4-1T1: Sprouty Receptor Tyrosine Kinase Signaling Antagonist 4-Intronic Transcript 1; TACE: transarterial chemoembolization; TGR5: G-protein-coupled bile acid receptor (Gpbar1); TNM: (Tumor, Node, Metastasis Staging System); tAFP: tumor alpha-fetoprotein; TSPYL5: testis-specific Y-encoded-like protein 5; ULK1: UNC51-like kinase 1; US: ultrasound; Y90: Yttrium-90 transarterial radioembolization.
